# Q-RPL: Q-Learning-Based Routing Protocol for Advanced Metering Infrastructure in Smart Grids

**DOI:** 10.3390/s24154818

**Published:** 2024-07-25

**Authors:** Carlos Lester Duenas Santos, Ahmad Mohamad Mezher, Juan Pablo Astudillo León, Julian Cardenas Barrera, Eduardo Castillo Guerra, Julian Meng

**Affiliations:** 1 Electrical and Computer Engineering Department, University of New Brunswick, Fredericton, NB E3B 5A3, Canada; ahmad.mezher@unb.ca (A.M.M.); jcardena@unb.ca (J.C.B.); eduardo.castillo@unb.ca (E.C.G.); jmeng@unb.ca (J.M.); 2 School of Mathematical and Computational Sciences, Yachay Tech University, Urcuquí 100115, Ecuador; jastudillo@yachaytech.edu.ec; 3 Communication Networks and Intelligent Services Research Group (ComNet Innova YT), Yachay Tech University, Urcuquí 100115, Ecuador

**Keywords:** machine learning, reinforcement learning, smart grids, routing protocol for low-power and lossy networks (RPL)

## Abstract

Efficient and reliable data routing is critical in Advanced Metering Infrastructure (AMI) within Smart Grids, dictating the overall network performance and resilience. This paper introduces Q-RPL, a novel Q-learning-based Routing Protocol designed to enhance routing decisions in AMI deployments based on wireless mesh technologies. Q-RPL leverages the principles of Reinforcement Learning (RL) to dynamically select optimal next-hop forwarding candidates, adapting to changing network conditions. The protocol operates on top of the standard IPv6 Routing Protocol for Low-Power and Lossy Networks (RPL), integrating it with intelligent decision-making capabilities. Through extensive simulations carried out in real map scenarios, Q-RPL demonstrates a significant improvement in key performance metrics such as packet delivery ratio, end-to-end delay, and compliant factor compared to the standard RPL implementation and other benchmark algorithms found in the literature. The adaptability and robustness of Q-RPL mark a significant advancement in the evolution of routing protocols for Smart Grid AMI, promising enhanced efficiency and reliability for future intelligent energy systems. The findings of this study also underscore the potential of Reinforcement Learning to improve networking protocols.

## 1. Introduction

The transformation of traditional electrical grids into Smart Grids (SGs) represents a major step forward in achieving efficient, reliable, and sustainable energy management. A key component of SGs is the Advanced Metering Infrastructure (AMI), which bridges the gap between electricity consumers and utilities. AMI is instrumental in automating meter reading processes and, more importantly, facilitating two-way communication that enables real-time data collection and analysis. This capability is crucial for enhancing demand response, where consumption patterns are adjusted in response to grid conditions, thus optimizing energy use and reducing costs. Consequently, the efficiency and reliability of the communication network supporting AMI are vital, as any disruption could potentially lead to disturbances or inefficiencies in energy distribution and management.

Utilization of wireless technologies has become popular in AMI deployments, offering flexibility and scalability. A distinguishing characteristic of some of those wireless technologies is the capacity to form mesh networks. Two standards have been relevant in this domain, IEEE 802.11s [1] and IEEE 802.15.4g [2]. In particular, the latter is the base standard for Wireless Smart Metering Utility Networks (Wi-SUN) [3] and was created to establish common and consistent communication specifications for utilities deploying SGs [4].

In wireless mesh networks, nodes transmit their own data and also serve as routers, forwarding data across the network. This brings forth the challenge of efficient and reliable data routing, a critical factor for the operational success of mesh networks. The main routing protocols utilized in AMI deployments reported in the literature are: the Geographic Routing Protocol (GPSR) [5], the Collection Tree Protocol (CTP) [6], the Optimized Link State Routing Protocol (OLSR) [7], the Routing Protocol for Low Power and Lossy Networks (RPL) [8], the Hybrid Routing Protocol (HYDRO) [9], the Lightweight On-Demand Ad hoc Distance-vector Routing Protocol–Next Generation (LOADng) [10], and the Hybrid Wireless Mesh Protocol (HWMP) [11].

Among these protocols, RPL holds a distinct position due to its designed characteristic to optimize data routing in low-power and lossy networks (LLNs), such as those found in AMI deployments [12]. Its suitability for being used in this kind of network has been covered in previous studies [13,14,15] which have compared RPL to other routing protocols.

Despite its advantages, RPL faces challenges in dynamic and diverse AMI environments. Previous works such as [16,17] have reflected the main limitations of RPL. One significant challenge is optimizing the parent selection process, which is crucial for ensuring efficient and reliable data transmission. The standard RPL mechanisms can lead to suboptimal routing decisions, affecting overall network performance, especially in dynamically changing environments typical of AMI deployments. Consequently, the problem of how a node selects the best candidate node to send or forward a packet among all possible alternatives remains an open research issue that continues to attract significant attention from the research community. This work contributes to addressing this problem by proposing Q-RPL, a novel approach to enhance RPL’s parent selection using Q-learning, a Reinforcement Learning (RL) technique.

Our motivation for using RL is built upon acknowledging that Machine Learning (ML) models have demonstrated effectiveness, as evidenced in previous studies [18,19], where ML-integrated RPL outperforms traditional routing protocols in Smart Grid networks. However, recent findings in [20] indicate that the performance of supervised ML models declines when applied to an AMI scenario different from its training environment. This limitation hinders model transferability between scenarios, and this is where Q-learning becomes a promising solution. Since it does not require datasets for training, successful implementation of Q-learning within RPL implies limitless applicability for this innovative approach across various AMI deployments.

To assess the effectiveness of our proposed approach, we conducted simulations using actual smart meter locations in the cities of Montreal and Barcelona, with traffic patterns representative of Smart Grid applications. Our new RL-based routing mechanism provided notable improvements in the packet delivery ratio (PDR), the end-to-end delay, and the compliant factor compared to the standard RPL implementation as well as to three other routing algorithms RPL+, ML-RPL, and Rl-RPL. The comparison was made across a range of traffic loads and in different deployments.

### Contribution and Organization

We highlight the following key contributions based on our findings in this paper:

- We propose a novel routing strategy based on a Reinforcement Learning technique, Q-learning, to improve the routing decisions of RPL in AMI deployments.
- Our approach balances the use of RL and traditional routing metrics, while the parent selection is guided by the Q-learning algorithm, traditional routing metrics like the Expected Transmission Count (ETX) and Received Signal Strength Indicator (RSSI) are used to enhance the Q-learning policy and the exploration-exploitation strategy.
- We have conducted simulations using smart meter locations from two real deployments of smart meters in the cities of Montreal and Barcelona to evaluate the performance of our proposed routing strategy and compare it to other benchmark protocols. The results show a significant improvement in key performance metrics such as PDR, average end-to-end delay, and compliant factor.
- Q-RPL bridges the gap between traditional routing methods and advanced Machine Learning techniques, offering insights into how these two domains can be effectively combined for improved network performance and reliability. Our approach, although used here with RPL, could potentially be adapted to other routing protocols used in the same context.

The rest of the paper is organized as follows. Section 2 discusses some related works. Section 3 provides a background to RPL. The design of the proposed solution is presented and described in detail in Section 4. The performance of the new routing strategy is evaluated and compared to other routing protocols in Section 5. Section 6 provides a technical and critical analysis of the new routing protocol along with some recommendations for its deployment. Lastly, Section 7 addresses the conclusions and future work.

## 2. Related Work

RPL was initially defined in RFC 6550 [8] and further shaped by related RFCs such as RFC 6551 [21], RFC 6552 [22], and RFC 6719 [23], which form the core of its functionality. To this day, RPL continues to capture the attention of the scientific community, as evidenced by the following recent studies. These studies reflect ongoing efforts to enhance and adapt RPL’s capabilities to meet the evolving demands of current communication networks.

Some authors have attempted to improve RPL by exploiting RPL’s capability to work with multiple instances. In the context of RPL, multiple instances refer to the capability of the protocol to support more than one distinct routing topology within the same physical network. This feature allows for different routing strategies or criteria to be applied simultaneously for different types of traffic or network conditions. A recent study [24] delved into deploying multiple RPL instances within Wireless Sensor Networks (WSNs). This approach, however, relied on the unmodified standard RPL implementations. A hop-count-based implementation was used for periodic and non-critical traffic, as well as the ETX-based implementation for critical data traffic.

Similarly, the research presented in [25] leverages the multi-topology routing capability of RPL to address Quality of Service (QoS) needs for various traffic types. This study introduces a novel parent selection framework using a multi-attribute decision-making method. It aims to overcome the limitations of RPL’s single metric approach, demonstrating improved QoS through the multi-topology strategy. Nonetheless, the simulation time used in this work raises questions about the ability to fully assess the long-term effectiveness and adaptability of the proposed solution across diverse network conditions.

A further study [26] introduces QWL-RPL, a variant of RPL designed to perform under heterogeneous traffic patterns. This protocol enhancement incorporates a queue and workload-based mechanism for routing decisions. The queue condition is determined by the queue length, i.e., the total number of packets waiting in the queue. In contrast, the workload is assessed by counting the packets transmitted at the MAC layer over set intervals. These metrics serve as indicators of network congestion and traffic load, respectively. Consequently, a node selects its preferred parent based on these criteria, favoring those with lower congestion and lighter traffic loads. Even though performance improvements are observed when compared to the standard RPL implementations, the omission of link quality metrics in the routing decision process could pose a significant drawback. By focusing solely on queue length and workload, there is a potential to overlook the link quality between nodes. This oversight could lead to scenarios where a node with lower packet transmissions and less congestion is selected as a preferred parent, despite having a poor link quality.

The authors of [27] tried to improve RPL performance by tackling the load balancing problem. They proposed Weighted Random Forward RPL (WRF-RPL). This variant of RPL combines the energy remaining in the nodes and their corresponding number of parents. These two routing metrics are the base of a weighted random selection algorithm used to choose the best next-hop candidate. WRF-RPL demonstrates improvements in network lifetime and PDR but ignores other important routing metrics related to link quality such as the ETX and RSSI. This could lead to choosing suboptimal routing paths, characterized by higher packet loss rates and reduced overall network reliability. In addition, considering only the number of parents may not accurately reflect the actual load on a node. For instance, nodes with fewer parents could be facing higher traffic loads or processing demands, contrary to those with a larger number of parents that might be underutilized. This approach risks creating an uneven distribution of network load, potentially diminishing network efficiency and performance.

The author of [28] proposed the RMP-RPL algorithm with a view to improving RPL reliability for critical applications. This paper proposes a new ranking calculation and parent selection method for RPL. In RMP-RPL, each node forwards its data packet to *n* number of ranked nodes based on three routing metrics: node mobility, alternative parent connectivity, and ETX. Despite the good results shown in this work in terms of PDR, sending packets to multiple nodes simultaneously can significantly increase the overall network traffic, and lead to severe congestion, especially in networks with limited bandwidth or a high number of nodes. This is a concern since the proposed method was not tested for different traffic loads.

The use of fuzzy logic to improve RPL has been considered by some authors in previous studies such as [29,30,31]. The authors of the most recent work [31] introduce a modified version of RPL named FL-HELR-OF. They propose a cross-layer architecture and a fuzzy logic system that integrates four input metrics: hop count, energy consumption, latency, and RSSI. The best route to reach DODAG’s root is chosen based on the output of the fuzzy system, while the new protocol outperforms the standard RPL implementations for different network sizes that range from 10 to 100 nodes, the use of logic fuzzy encloses complex configuration and tuning of membership functions and rule sets.

The problem of routing overhead in RPL is addressed in [32]. The authors propose an adaptive routing algorithm, named Tabu-RPL, that dynamically adjusts data dissemination paths based on network conditions and device capabilities. The core of the proposal is the integration into RPL of the Tabu Search algorithm, which is a metaheuristic search method used to solve mathematical optimization problems. Tabu-RPL achieves a 30% reduction in network overhead according to the results presented in the paper. It is worth mentioning that the design basically focuses on optimizing the parent selection considering metrics such as ETX and residual energy, however, the paper does not clearly delineate how this approach translates into reduced network overhead. Additionally, the design suggests that the algorithm considers only dynamic adjustments during the search process phase since there is no information about how the algorithm continues to look for better neighbors after the stopping condition is reached. This aspect can potentially affect the adaptability of the protocol in more changing environments.

A further study [33] addresses the issue of energy efficiency in RPL. The authors propose a modification to the traditional trickle timer algorithm, introducing an enhanced version named EE-trickle. The primary aim is to reduce energy consumption and improve the PDR of the network. The standard trickle algorithm, which controls the timing and dissemination of control messages in RPL can lead to high energy consumption due to extended listening periods and frequent transmissions. The EE-trickle algorithm addresses this by optimizing how listening and transmission intervals are managed, thereby reducing unnecessary energy expenditure. The performance evaluations demonstrate that EE-trickle significantly lowers energy usage per node and better PDR is achieved in the network compared to when the standard approach is used, while the results are encouraging in terms of power consumption and PDR, solely managing control messages in this manner may overlook other critical factors such as link quality variability that can impact network reliability and efficiency.

A recent trend in improving RPL has been to use Machine Learning to enhance routing decisions. For example, in [34], RPL+ introduces a refined parent selection strategy to choose the best forwarding node when two or more candidates have the same ranking in the RPL destination-oriented routing tree. Central to RPL+ is the utilization of a Random Forest (RF) algorithm [35] for analyzing the significance of different routing metrics. These metrics include the ETX, MAC layer losses, channel utilization, and throughput. Based on the assessed importance of each metric, weights are assigned within a forwarding score function, facilitating the identification of the most suitable forwarding node among available candidates. The main limitation of this proposal is the static weight assignment, which can restrict its flexibility and responsiveness to varying network load conditions. That said, the simulation results show that RPL+ achieves a notable improvement in PDR, outperforming standard RPL implementations across various network sizes.

The authors of [18] proposed ML-RPL. This research explores the potential of ML to enhance wireless communication networks, specifically in the context of Smart Grids. This proposal integrates CatBoost, a Gradient Boosted Decision Trees (GBDT) algorithm, into RPL to optimize routing decisions. The final ML model was trained and optimized on a dataset of routing metrics obtained from many simulation campaigns, considering a real deployment of smart meters in the city of Montreal. Each smart meter uses the ML model to predict the probability of successfully reaching a destination node, and then the candidate node with the highest probability of effectively being reached is chosen as the preferred next hop. ML-RPL significantly improved the PDR compared to a standard RPL implementation and RPL+.

Another ML-based variant of RPL is presented in [19] based on the dataset obtained in [18]. In this case, the authors used the Gaussian Naive Bayes algorithm and integrated it into RPL, resulting in GNB-RPL. The main goal was to take advantage of the benefits of this ML algorithm that the authors considered particularly relevant in SG wireless communication scenarios such as simplicity, scalability, and reduced training data requirements.

In [20], is exposed the main limitation of the previous two studies: both Catboost and Naive Bayes are supervised ML methods, so their performance in making the best routing decisions will depend on the dataset on which they are trained. Therefore, a model trained and optimized for a particular scenario could underperform significantly in another type of scenario, as is shown in [20].

A potential solution to avoid the need for frequent retraining or updates to adapt the supervised ML models to changing network scenarios, which can be impractical in dynamic AMI environments, is the application of Reinforcement Learning. RL has been gaining attention in various routing contexts as shown in [36,37,38]. For the applicability of this technique on RPL or any other routing protocol utilized in AMI networks, the following works are of special interest.

The research presented in [39] introduces a Reinforcement-Learning-based routing protocol for WSNs. The protocol aims to choose the best parent node in a tree topology by using Q-learning. According to the simulation results shown in the article, the proposed method outperforms two linear-weighted-sum-based parent selection algorithms, in terms of packet delivery ratio, end-to-end delay, and energy consumption. However, some aspects of the protocol design could benefit from further elaboration. For instance, a more detailed explanation of the reward function’s construction, including how various performance metrics are integrated, would enhance the clarity and robustness of the decision-making process. Additionally, the protocol’s approach of utilizing periodic hello messages every 5 s may pose scalability challenges as network size increases. Exploring adaptive strategies for the frequency of these messages, similar to the trickle timer algorithm used in RPL, could potentially optimize network efficiency and scalability. Lastly, adding concerns to the overall network efficiency is the protocol’s cycle detection mechanism, which requires each node to send a join request message containing its list of child nodes to prospective parent nodes.

In a direct attempt to incorporate RL into RPL, the authors of [40] introduce a new RPL variant for Internet-of-Things (IoT) environments, where the parent selection relies on the Q-learning algorithm. The article addresses critical challenges of RPL such as the negative impacts of instantaneous path selection and the need to consider the dynamic conditions of nodes for parent selection. The approach demonstrates improvements in delivery rates, latency, and energy consumption compared to existing methods as shown through simulation tests. Nevertheless, there are some important considerations regarding the design and implementation that raise concerns and merit closer scrutiny. For instance, the authors assign equal weights to all the metrics in the reward function, which does not accurately reflect their relative importance or interdependencies. In addition, we agree that maintaining a stable parent after the algorithm converges, as the authors do, can enhance the consistency and reliability of the routing path. However, this stability might also reduce the protocol’s responsiveness to sudden changes in network conditions after convergence.

Another study where RL was used along with RPL is [41]. The study introduces QFS-RPL, a novel RPL-based routing protocol enhanced by the Q-learning algorithm and concepts from the Fisheye State Routing protocol. An important element in QFS-RPL’s design is the modification of the traditional Q-function by incorporating additional parameters that reflect the state of the network more comprehensively. According to the results presented in the paper, QFS-RPL is particularly suited for scenarios where nodes are mobile. Conversely, in networks with static nodes, QFS-RPL performs similarly to the standard RPL.

Lastly, an adaptive control of transmission power for RPL, named ACTOR, is presented in [42]. This article introduces a dynamic approach to optimizing transmission power to improve throughput in dense networks. ACTOR extends the standard RPL by integrating a specific RL strategy, the Upper Confidence Bound (UCB), to efficiently manage the exploration and exploitation trade-off. This method allows for passive exploration of different transmission power levels, aiming to enhance network performance by dynamically adjusting power settings based on real-time conditions. Although ACTOR demonstrates positive results due to adjustments in transmit power, its main drawback is using only the ETX metric for routing decisions, which is similar to the standard RPL implementation.

In light of the comprehensive review of existing modifications and enhancements to RPL, it becomes evident that while significant progress has been made, particularly in employing ML-based techniques, there remain inherent limitations to these routing solutions. Table 1 summarizes the main aspects of the studies discussed in this section.

## 3. RPL Parent Selection Background

This section aims to provide the necessary background on RPL, as our design builds upon and enhances the RPL protocol. It lays the groundwork for understanding the enhancements introduced by our RL-based approach.

**Figure 1:**
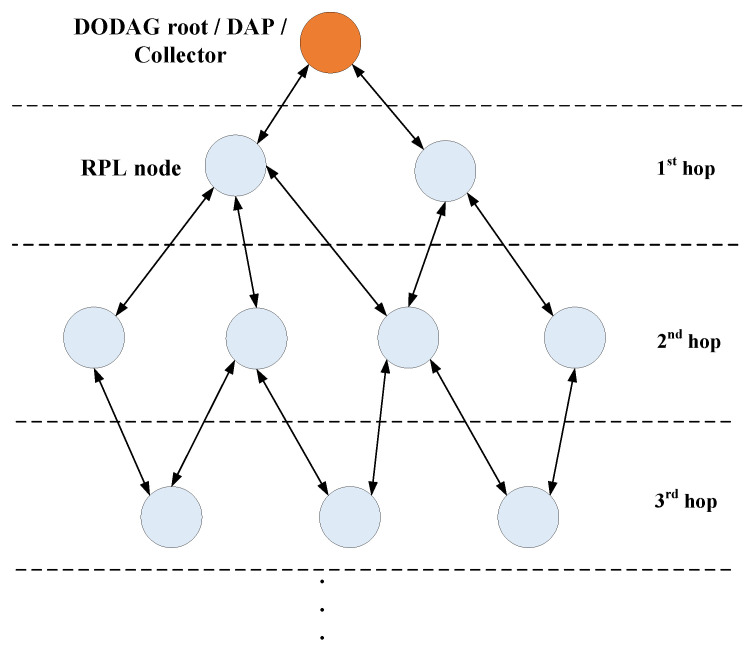
Hierarchical routing with RPL.

RPL is a distance vector routing protocol that is adapted to a variety of Low-Power and Lossy Networks (LLNs). The protocol constructs a destination-oriented directed acyclic graph (DODAG) [8], which is a multi-hop routing tree rooted at single root node, that in the context of AMI is usually named a data aggregation point (DAP) or simply collector. Figure 1 shows the routing hierarchy within RPL.

The root node initiates the network by transmitting a control message, called a DODAG Information Object (DIO), which includes rank information. Each node in the network, upon receiving the DIO, computes its ranking and selects a parent to form a loop-free topology. This makes RPL a highly scalable protocol that can accommodate the large and growing number of nodes in an AMI communication network.

The selection of parent nodes within the DODAG is governed by an Objective Function (OF). The OF is responsible for defining the criteria used to evaluate potential parent nodes, thereby influencing the route optimization and network dynamics. Two standardized OFs commonly used in RPL are the Objective Function Zero (OF0) [22] and the Minimum Rank with Hysteresis Objective Function (MRHOF) [23].

OF0 primarily uses hop count as its metric. It selects the parent node that offers the shortest path to the root, simplifying the routing process but potentially overlooking other critical metrics like link quality or reliability.

MRHOF is more sophisticated and can consider multiple metrics, primarily focusing on link quality and node stability. It uses a cost function that typically includes the ETX and a hysteresis component to prevent frequent changes in parent selection, which could destabilize the network.

Other solutions to the parent selection problem include a variety of methods as was shown in Section 2. The next section will provide a comprehensive explanation of our approach to tackle this problem. It will outline how our method works and highlight the distinctive elements that make it unique to other implementations.

## 4. Q-RPL: Q-Learning-Based Routing Protocol Design

**Figure 2:**
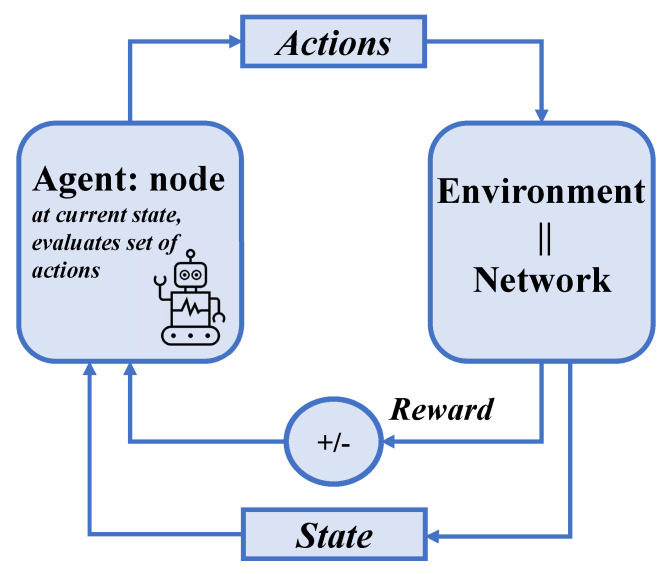
The interaction between the agent and its environment in the context of networking.

Reinforcement Learning [43] stands out as a powerful paradigm particularly suited for scenarios where an agent must make decisions based on interactions with a dynamic environment. Distinguished by its ability to learn optimal actions through trial and error, RL enables agents to adapt their strategies over time, aiming to maximize cumulative rewards. This approach is fundamentally different from traditional supervised learning, as it does not rely on a labeled dataset but instead learns from the consequences of actions taken in a given state. Central to RL is the concept of a learning agent that observes the state of the environment, takes actions, and receives feedback in the form of rewards or penalties as shown in Figure 2. This feedback helps the agent understand the effectiveness of its actions and guides it in refining its decision-making process. RL’s capacity to continually adapt and learn from ongoing interactions makes it particularly applicable to complex and changing environments, such as those found in network routing protocols. RL offers in this context a promising avenue for developing advanced routing protocols that can dynamically adjust to varying network conditions, thereby enhancing efficiency, reliability, and overall performance.

### 4.1. Q-Learning Algorithm

Q-learning is one of the most popular RL algorithms [44]. It is an off-policy learner that seeks to find the best action to take given the current state. It is known for its simplicity and effectiveness, especially in environments with a finite number of states and actions. In Q-learning, a Q-function is used to measure the quality of a state–action pair, based on the observed reward. The following equation shows how the Q-values are updated when action *a* is taken by a node *i* in state *s*, yielding a reward *r*.

(1) $$Q\left(s^{i},a^{i}\right)\leftarrow \left(1-\alpha \right)Q\left(s^{i},a^{i}\right)+\alpha \left[r+\gamma \max_{a^{j}}Q\left(s^{j},a^{j}\right)\right]$$

where $Q(s^{i},a^{i})$ is the value of the current state–action pair, α∈[0,1] is a parameter called learning rate, *r* is the reward received after taking action *a*, γ∈[0,1] is another parameter named discount factor, and $\max_{a^{j}}Q\left(s^{j},a^{j}\right)$ is the maximum reward expected from all the possible actions at the next hop candidate *j*.

The learning rate determines how much new information affects the existing Q-value. A higher learning rate allows the model to adjust more quickly to changes but can cause the learning process to be unstable. Meanwhile, the discount factor balances the importance of immediate and future rewards. A higher discount factor makes the agent more forward-looking by emphasizing the potential future benefits of current actions.

Other key elements in the design of any RL solution based on the Q-learning algorithm are the formulation of the state–action space, reward, and policy update. The next subsections provide more details about these specific components in our design solution for routing in AMI.

### 4.2. State–Action Space Design

The definition of state(s) and action(s) is crucial in Reinforcement Learning, particularly when applied to routing in network environments. The state represents the current configuration of the network, and the action signifies the possible decisions a node (an RL agent) can make. We observed in Section 2 that existing studies such as [39,40] have formulated states using routing metrics from neighboring nodes. Although this approach is intuitive, it introduces significant challenges, particularly in the dimensionality of the state space. If the routing metrics are continuous or exhibit high variability, discretizing the state space becomes necessary to prevent an unmanageably large Q-table. The size of the state space increases with each added metric. For instance, if each of *m* metrics can take *x* values (either because they are inherently discrete or were discretized), the total state space size would be $x^{m}$. Subsequently, the state-action space size would be determined by multiplying the state space size by the number of potential actions available at each state. This requires careful consideration, especially for resource-constrained devices.

In our protocol design, we conceptualize states differently. Here, states represent the destination nodes that a node aims to reach, typically the DODAG root in RPL. So, for node *i*, the state $s^{i}\in S^{i}=\left\{s_{1}^{i},s_{2}^{i},...,s_{n}^{i}\right\}$, where *n* represents the number of destination nodes in the network. This approach is particularly efficient when nodes operate in non-storing mode, where all traffic is directed towards the DODAG root, significantly simplifying the state space. Actions in our model correspond to the choice of the next hop to reach the destination. Thus, actions for a node *i* can be expressed as $a^{i}\in A^{i}=\left\{a_{1}^{i},a_{2}^{i},...,a_{k}^{i}\right\}$, where *k* is the number of possible next-hop candidates for this node. Therefore, the dimension of our Q-table, which has a Q-value for each pair of state–action, is proportional to the size of the neighbor table. This design not only addresses the dimensionality and discretization issues but also aligns well with the operational modes of RPL. Table 2 shows a typical Q-table for a node *i*, where the Q-value for each entry in the Q-table is calculated with the Q-learning formula introduced in Equation (1).

### 4.3. Reward and Policy Design

The reward (r) is another important component in the Q-learning algorithm. Reward refers to the feedback that the agent (in this case, a network node) receives after taking a specific action. This feedback is crucial as it guides the learning process, shaping the node’s understanding of which actions are beneficial and should be repeated in the future.

We consider that the reward function in the context of routing protocols must be designed to reflect the effectiveness of routing decisions in the most direct way. Different from previous studies, such as [39,40], that assigned weights to distinct indirect performance metrics (number of hops, ETX, congestion status, etc.) to estimate the performance of taking an action, our reward function is assigned based on the success and efficiency of packet transmissions to the next hop. Thus, we take into account the number of transmission attempts required to successfully deliver a packet. Consequently, the structure of the reward is as follows:

- **First attempt success.** A reward of +2 is granted for a packet that successfully reaches its next hop on the first attempt, not requiring re-transmissions. This scenario represents the ideal case, where the routing decision leads to an efficient and effective outcome.
- **Success on the first re-transmission.** After a failure on the first transmission attempt, if the packet is successfully transmitted on the first re-transmission attempt, the reward is set to +1. This represents a less optimal successful transmission.
- **Success on the second re-transmission.** A reward of 0 is given for a packet that reaches the next hop on the second re-transmission. This situation indicates a less efficient routing decision.
- **Success on the third re-transmission.** A reward of −1 is given for a packet that reaches the next hop on the third re-transmission. This situation warrants a slight penalty.
- **Failure to transmit.** If the packet fails to reach the next hop after all attempts, the reward is −2. This condition represents an action failure and is penalized accordingly.

Differentiating between the varying levels of transmission efficiency ensures that the learning process inherently favors routing decisions that lead to successful and efficient packet deliveries. The negative reward for failed transmissions further reinforces the need for reliable routing choices. Over time, this mechanism allows the Q-learning algorithm to discern and prefer routing paths that not only succeed in delivering packets but do so with optimal efficiency.

For instance, consider a dynamic scenario in which different levels of signal interference or congestion can affect transmission success and where a node must choose between two potential candidates for routing data packets to a destination. Option A is a node that often experiences signal interference due to its location, while option B benefits from a clear and unobstructed link. Initially, the node may randomly select between these options, but will evaluate the number of re-transmissions required to successfully deliver a packet. When sending packets via option A, three re-transmissions are typically necessary, each resulting in a reward of −1. In contrast, choosing option B usually requires no re-transmissions, yielding a reward of +2. Given the operation of the Q-learning algorithm, this reward system will naturally favor option B in the long run.

Furthermore, in designing the reward system, we have considered the compatibility of our reward design with the wireless communication standard in the physical and MAC layers. The target of three re-transmissions aligns with the default number of re-transmissions set by the IEEE 802.15.4 standard, as detailed in [45].

In summary, if we denote the number of re-transmission attempts to successfully transmit a packet by *m*, the reward function can be expressed as Equation (2):

(2) $$r=\left\{\begin{array}{cc} 2-m, & \mathrm{for}\;0\leq m\leq 3\\ -2, & \mathrm{if}\;\mathrm{the}\;\mathrm{packet}\;\mathrm{fails}\;\mathrm{after}\;\mathrm{all}\;\mathrm{attempts}. \end{array}\right.$$

With the reward structure defined, the next critical element in our Q-learning-based routing protocol is the policy, which dictates how decisions are made based on the learned Q-values. The policy, that we define as π(s), is a strategy that each node employs to decide which action to take in a given state. It plays an important role in balancing the exploration of new routing paths against the exploitation of the best-known action. A balance that is crucial for the adaptability and effectiveness of any routing protocol.

We adopt the ϵ-greedy policy, a popular choice in RL tasks for its simplicity and effectiveness. With probability ϵ, the policy allows the routing node to choose randomly a next-hop candidate in the Q-table, encouraging the discovery of a potentially better candidate. Conversely, with a probability of (1−ϵ), the node exploits its accumulated knowledge by choosing the candidate with the highest Q-value for a given state *s*. The ϵ-greedy policy for a node *i* at state $s_{1}^{i}$ can be represented as follows:

(3) $$\pi \left(s_{1}^{i}\right)=\left\{\begin{array}{cc} a^{i}∼\mathrm{Uniform}\left(A^{i}\left(s_{1}^{i}\right)\right) & \mathrm{with}\;\mathrm{probability}\;\epsilon ,\\ \underset{a^{i}\in A^{i}}{\arg\;\max}\;Q\left(s_{1}^{i},a^{i}\right) & \mathrm{with}\;\mathrm{probability}\;1-\epsilon . \end{array}\right.$$

where the term $a^{i}∼\mathrm{Uniform}\left(A^{i}\left(s_{1}^{i}\right)\right)$ indicates that the action $a^{i}$ is chosen randomly following a uniform distribution over the set of all possible actions available at the current state.

A common practice in RL that we follow in our design is to dynamically adjust ϵ over time. Starting with a higher ϵ and gradually decreasing it, allows for more exploration early in training and more exploitation later on. Furthermore, to prevent the model from becoming too exploitative, we establish a lower bound for ϵ, ensuring that the nodes will always maintain some degree of exploration. This dynamic adjustment of ϵ is essential for adapting to the changing network conditions and continuously refining routing decisions as the learning algorithm accumulates more knowledge and experience. Algorithm 1 summarizes the operation of the ϵ-greedy policy.

**Algorithm** **1** ϵ-Greedy Policy.**Require:** $Q(s^{i},a^{i})$
 Initialize: ϵ, $\epsilon_{min}$, $\epsilon_{decay}$

 **while** the learning process is ongoing **do**

  Choose a random number *x*, x∈[0,1]
  **if** x<ϵ **then**

   Choose a random action $a^{i}$:

   $\pi \left(s^{i}\right)=a^{i}∼\mathrm{Uniform}\left(A^{i}\left(s^{i}\right)\right)$

  **else**

   Choose the best-known action $a^{i}$:

   $\pi \left(s^{i}\right)=\underset{a^{i}\in A^{i}}{\arg\;\max}\;Q\left(s^{i},a^{i}\right)$

  **end if**

  Decrease ϵ gradually: $\epsilon \leftarrow \max(\epsilon \times \epsilon_{decay},\epsilon_{min})$

 **end while**


### 4.4. Integration into RPL

**Figure 3:**
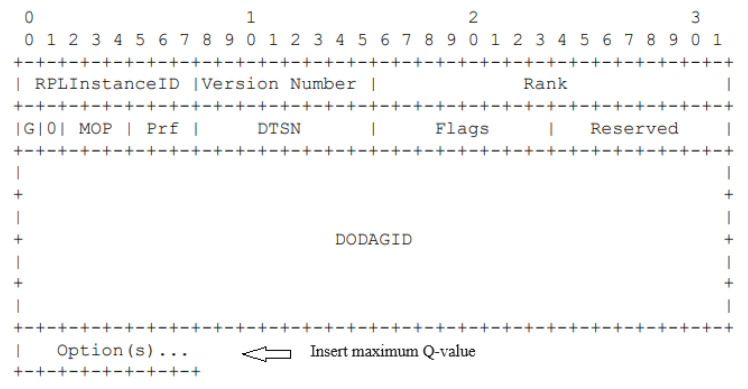
DIO message base format.

The integration of Q-learning into the RPL protocol represents a significant advancement in routing decision-making for AMI. The Q-learning algorithm in our enhanced RPL framework specifically assists in the parent selection process. Each node employs the learned Q-values to make informed decisions on which next hop to choose as its preferred parent for routing packets toward the root according to the policy defined in Section 4.3. This approach leverages the continuous learning capability of Q-learning to dynamically adapt to changing network conditions, thereby optimizing the routing paths over time. We keep the core of RPL functionalities like ranking calculation based on RFC 6550, trickle timer algorithm, loop detection mechanism, and the use of signaling messages such as DIO, Destination Advertisement Object (DAO), and DODAG Information Solicitation (DIS). However, to facilitate the learning-based approach, we propose a modification in the DIO messages. Each node, in addition to the standard RPL information, broadcasts its maximum Q-value. This modification allows neighboring nodes to be aware of the routing efficacy (as measured by the Q-values) of their potential parents and update the Q-learning equation accordingly. The maximum Q-value is included in the DAG Metric Container in the option field of the DIO messages, as shown in Figure 3.

**Figure 4:**
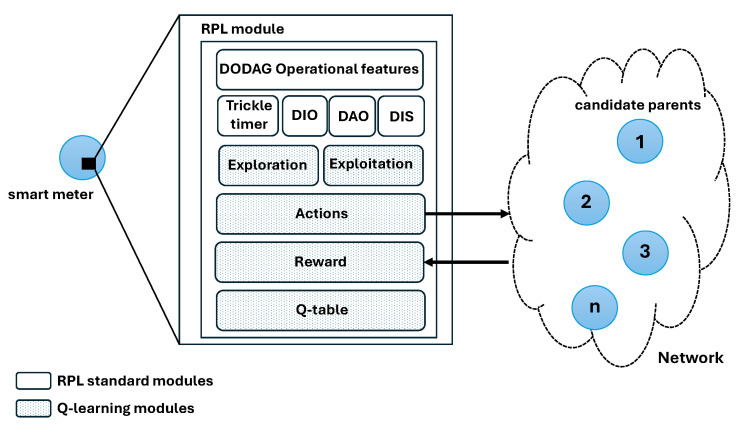
Architecture of the Q-RPL algorithm.

Figure 4 illustrates the Q-RPL architecture after integrating the Q-learning modules into RPL. It shows the main modules of RPL alongside the Q-learning modules in the same framework. RPL modules are responsible for handling DODAG formation, maintaining network structure, and facilitating basic communication among nodes. In contrast, the Q-learning modules are tasked with optimizing parent selection.

Another important difference in our design with respect to previous studies is the role of the classic routing metrics such as the ETX and RSSI, while the Q-learning algorithm plays the primary role in the decision-making mechanism, the ETX and RSSI serve as a policy enhancement and for guiding exploration, respectively.

In cases where two potential parents have identical Q-values, the ETX metric is used as a tiebreaker. Since the ETX represents the number of transmissions expected to deliver a packet over a link successfully, preference is given to the parent with a lower ETX, indicating a more reliable link. The metric is calculated according to the following expression:

(4) $$ETX=\frac{1}{Df\cdot Dr}$$

where Df is the measured probability that a packet is received by the neighbor and Dr is the measured probability that the acknowledgment packet is successfully received [21]. The metric is assessed after the transmission of actual data and acknowledgment within the re-transmission mechanism at the MAC layer. Thus, it is updated when a packet is successfully acknowledged or when the maximum number of re-transmissions is exceeded. The ETX is also smoothed by using an exponential weighted moving average (EWMA) filter following best practices:

(5) $$ETX_{new}=\lambda \cdot ETX_{current}+\left(1-\lambda \right)\cdot ETX_{prior}$$

where the value of λ is implementation dependent, and it has been set to 0.8 as in other implementations [46].

Furthermore, we introduce an exploration trigger based on RSSI variations, a critical metric in ensuring optimal routing decisions, as previously discussed in [18]. To this end, each smart meter actively monitors the RSSI value of its neighboring nodes. If the average of the RSSI of one of the neighbors drops below a threshold, the node is not considered in the exploration phase. This way, the algorithm avoids wasting time exploring nodes with poor link quality. In the case that the node with a declining link quality is the current preferred parent, selected based on its high Q-value during the exploitation phase, the algorithm triggers a reassessment of the parent choice, prompting exploration. We have established a threshold of 10% below the smart meters’ receiver sensitivity as a reasonable indicator of a poor link quality. This dynamic response to changing link qualities, facilitated by the integration of RSSI monitoring, significantly boosts the algorithm’s capability to adapt and maintain effective routing paths in varying network conditions.

Algorithm 2 presents a pseudocode that outlines each step of the enhanced parent selection process for RPL. Starting with how the next-hop node is chosen based on the ϵ-greedy policy, followed by the reward calculation, the update of the Q-values, and finally, the monitoring mechanism to detect nodes with decaying link quality.
**Algorithm** **2** Enhanced Parent Selection Algorithm for RPL.**Require:** K, a set of *n* candidate parents of node *i*, where $P_{k}$ is the current_preferred_parent. $Q(s^{i},a^{i})$. **Initialize:**
ϵ, $\epsilon_{min}$, $\epsilon_{decay}$, α, γ, threshold. **for** each new routing decision **do**  **Choose next-hop based on ϵ-greedy policy:**  Choose a random number *x*, x∈[0,1]  **if** x<ϵ **then**   Choose a random action $a^{i}$:   $\pi \left(s^{i}\right)=a^{i}∼\mathrm{Uniform}\left(A^{i}\left(s^{i}\right)\right)$   $P_{k}\leftarrow \pi \left(s^{i}\right)$  **else**   Choose the best-known action $a^{i}$:   $\pi \left(s^{i}\right)=\underset{a^{i}\in A^{i}}{\arg\;\max}\;Q\left(s^{i},a^{i}\right)$   **if** tie in Q-values **then**    Identify all actions $a^{i}$ with max Q-value: E    Choose the action with the lowest ETX value:    $\pi \left(s^{i}\right)=\underset{a^{i}\in E}{\arg\;\min}\;\mathrm{ETX}\left(a^{i}\right)$   **end if**   $P_{k}\leftarrow \pi \left(s^{i}\right)$  **end if**  **Transmit packet to chosen next-hop: $P_{k}$**  **Observe outcome and calculate reward:**  **if** packetfail **then**   r=−2  **else**   get → re-transmission attempt (m)   r=2−m  **end if**  **Update Q-value for the state–action pair:**  $Q\left(s^{i},a^{i}\right)\leftarrow \left(1-\alpha \right)Q\left(s^{i},a^{i}\right)+\alpha \left[r+\gamma \max_{a^{j}}Q\left(s^{j},a^{j}\right)\right]$  **Update ϵ:**
$\epsilon \leftarrow \max(\epsilon \times \epsilon_{decay},\epsilon_{min})$ **end for** **Monitor RSSI of nodes in K:** **if**
$RSSI_{k}$ < threshold, k∈K **then**   **if** ϵ == $\epsilon_{min}$ AND *k* == $P_{k}$ **then**    trigger exploration by resetting ϵ to initial value   **else**    remove *k* from K   **end if** **end if**

## 5. Performance Evaluation

We present the performance evaluation of our proposed Q-RPL in this section. We compare its effectiveness against a standard RPL implementation (MRHOF) as well as three other routing solutions previously discussed in the related work section: RPL+ [34], ML-RPL [18], and Rl-RPL [40]. To ensure a comprehensive analysis, we utilize two distinct scenarios based on actual deployments in the cities of Montreal and Barcelona. These scenarios vary in the number of nodes and the network topology.

The next subsection outlines the specifics of our simulation settings, followed by subsections presenting the results obtained from each scenario and another subsection with a general discussion of the results.

### 5.1. Simulation Settings

The simulations are carried out using the discrete network simulator OMNeT++ [47]. OMNeT++ is one of the most widely used and powerful simulation tools for network modeling [48]. The simulator works under an Eclipse-based IDE, and it can run basically on all platforms where a C++ compiler is available (Linux, Mac OS/X, Windows). It is free for academia and open source, which allows the reuse and modification of its modules. In addition, several simulation frameworks have been created to extend the functionality of the simulator to specific areas. One of those frameworks is INET, which we have used along with OMNeT++ since it includes all the protocol layers that we need to create our simulation environment. In comparison to other network simulator alternatives like ns-3 [49], RPL implementations available in OMNeT++ are more mature and updated than those available in ns-3 [50,51,52]. Lastly, while in [53], there is a mention of an implementation of IEEE 802.15.4g/Wi-SUN for the ns-3 simulator, to the best of our knowledge, this implementation is not available. All the previous reasons make OMNeT++ a suitable simulator to ensure an accurate and comprehensive evaluation of our proposed solution.

**Figure 5:**
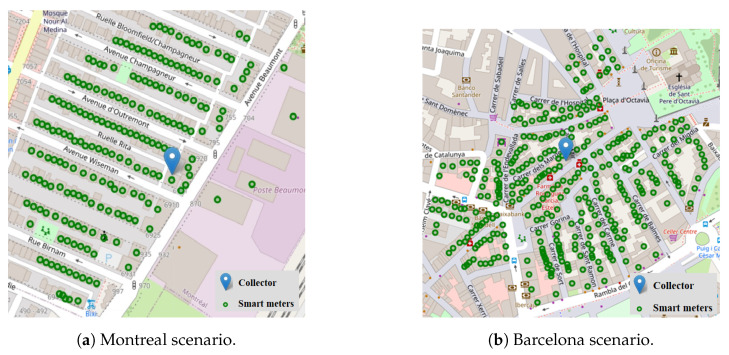
Urban scenarios.

The first scenario in which our proposal is tested consists of a deployment of 200 smart meters and one collector in the city of Montreal, while the second scenario in the city of Barcelona has 355 smart meters and also one collector. This larger scenario allows us to test the scalability and efficiency of Q-RPL in a more demanding environment, where the increased number of smart meters and potential network congestion present additional challenges. The scenarios are depicted in Figure 5a,b. The channel characteristics, physical-MAC layer, and learning parameters are shown in Table 3.

The traffic load is varied in each scenario according to Table 4. The applications that we have considered are typical in Smart Grids: Meter Reading (MR), Alarm Events (AEs), and Power Quality (PQ). MR refers to the usage information that smart meters collect and must report periodically to utilities. AEs is the second application taken into consideration. Alarms can happen at any time and are sent randomly during the simulation time by a percentage of smart meters. AEs can include events such as measurement failure, system restart, system memory full, configuration errors, etc. The other application considered is PQ. Examples of PQ events include leading/lagging power measurements, imbalance in energy flow, voltage fluctuations, harmonics, and voltage sags and swells. For traffic load 1, MR is transmitted for each smart meter every hour, while AEs and PQ are transmitted by 25% of the smart meters in the scenario. In contrast, traffic load 2, while still including the same applications, shows a variation in the sending frequency for MR, which is now every 30 min. Moreover, the percentage of meters involved in transmitting alarm events and power quality data is increased to 50% under traffic load 2, as opposed to 25% in traffic load 1.

### 5.2. Montreal Scenario

**Figure 6:**
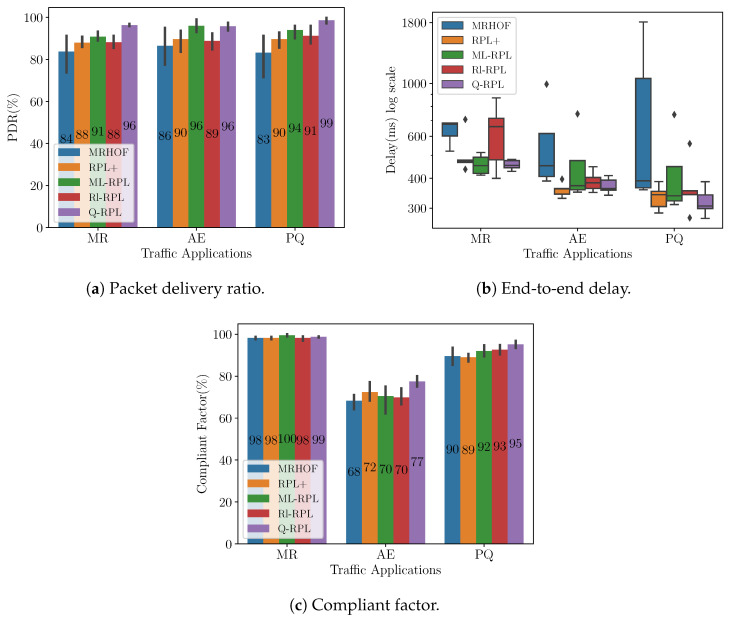
Performance metrics in the Montreal scenario under traffic load 1.

Figure 6a shows the PDR per application measured at the collector for traffic load 1 in the Montreal deployment. Recall that the PDR expresses the ratio of packets successfully delivered to the destination to those generated by the source. Specifically, in the MR application, Q-RPL exceeds the performance of MRHOF, RPL+, Rl-RPL, and ML-RPL by 12%, 8%, 8%, and 5%, respectively. In alarm events, Q-RPL matches ML-RPL’s performance while surpassing RPL+ by 6%, Rl-RPL by 7%, and MRHOF by 10%. The most significant disparity is observed in the PQ application, where Q-RPL achieves a 99% PDR, outperforming the next closest protocol, ML-RPL, by 5%, and surpassing Rl-RPL, RPL+, and MRHOF by 8%, 9%, and 16% respectively.

The distribution of the end-to-end delay of the packets that reach the destination is depicted in Figure 6b using a box plot representation. In this comparison, MRHOF exhibits the highest median delay across all traffic applications. Rl-RPL has the second-worst performance for MR application, but closely matches the median delay values of the other protocols for the AE and PQ applications. RPL+, ML-RPL, and Q-RPL have median delay values within 50 ms of each other across all the applications, however, the box plot reveals that RPL+ and Q-RPL have narrower Interquartile Ranges (IQRs) compared to ML-RPL. This characteristic indicates a more consistent and predictable delay performance of RPL+ and Q-RPL.

To complement the end-to-end delay analysis, we present the compliant factor (CF) in Figure 6c. The CF is the ratio of packets that not only successfully reach their destination but also do so within a predefined delay criteria specific to each application, according to Table 5. The metric is expressed in percentages and is particularly important in networks like Smart Grids, where different applications may have varying and stringent requirements for packet delivery times. The CF has been used in previous works such as [54,55] for comparing different Smart Grid communication technologies. In summary, the CF can be formulated as follows:

(6) $$CF=\left(\frac{\mathrm{Total}\;\mathrm{number}\;\mathrm{of}\;\mathrm{packets}\;\mathrm{meeting}\;\mathrm{the}\;\mathrm{delay}\;\mathrm{criteria}}{\mathrm{Total}\;\mathrm{number}\;\mathrm{of}\;\mathrm{successful}\;\mathrm{packets}\;\mathrm{received}}\right)\times 100\%$$

In the case of MR traffic, all the routing protocols under analysis achieve a CF close to 100%. However, Q-RPL displays a clear advantage in AE traffic, improving the CF by 9%, 7%, 7%, and 5% compared to MRHOF, ML-RPL, Rl-RPL, and RPL+, respectively. For PQ traffic, Q-RPL maintains the highest CF at 95%.

**Figure 7:**
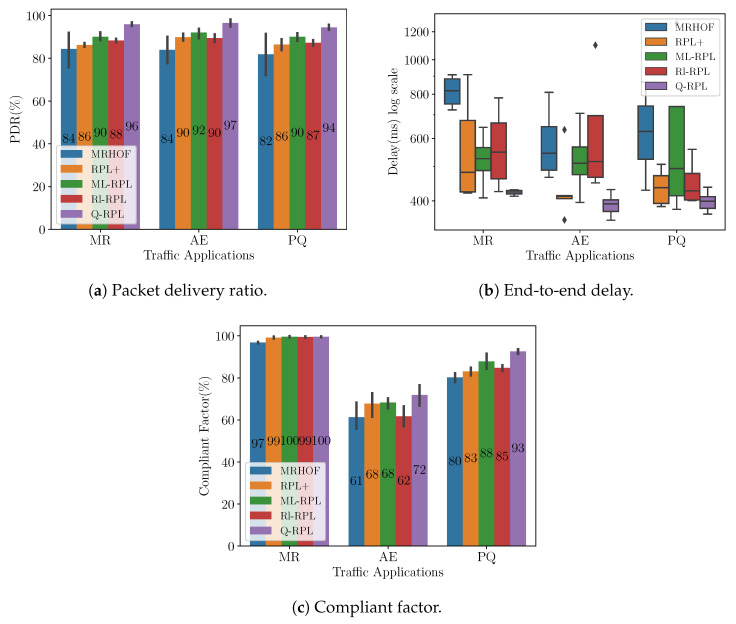
Performance metrics in the Montreal scenario under traffic load 2.

In order to assess the robustness of our novel routing protocol when network load increases, we decreased the sending interval of the MR application by 50% and doubled the percentage of the smart meters sending alarm reports and power quality events. Figure 7a shows the PDR achieved by each routing protocol under the new traffic load conditions. Based on the results, Q-RPL demonstrates superior performance across all three applications compared to MRHOF, RPL+, ML-RPL, and Rl-RPL. Notably, for the AE traffic, where Q-RPL previously matched ML-RPL’s performance, it now surpasses it by 5%, further demonstrating its effectiveness in handling increased network traffic.

Figure 7b presents the end-to-end delay under the new traffic conditions. Unlike the other routing protocols, which exhibited increased median delay values under higher traffic load, Q-RPL maintained a performance similar to lower traffic conditions, with the median delay hovering around 400 ms across all three traffic applications. In addition, the other protocols showed greater variability in packet delay times, contrasting it with the stability and consistency in the packet delay of Q-RPL.

Lastly, Figure 7c depicts the CF resulting from the increase in the traffic load. Similar to the outcomes observed with traffic load 1, all protocols perform well in terms of CF for MR traffic. However, a notable decrease in CF is observed for the AE and PQ applications compared to the first traffic load scenario. MRHOF and Rl-RPL experienced the most significant reduction. MRHOF dropped its CF by 7% and 10% for AE and PQ traffic, while Rl-RPL dropped its CF by 8% for both traffic applications. RPL+ and ML-RPL also saw declines in CF, though to a smaller degree, a 4% and 6% drop for RPL+ and a 2% and 4% CF decrease for ML-RPL in AE and PQ traffic, respectively. Notably, despite a 5% CF reduction in AE traffic, Q-RPL maintained the highest CF among all protocols for AE traffic. In terms of PQ traffic, Q-RPL achieved a CF of 93%, only a slight 2% decrease from traffic load 1, but a still significantly higher CF than MRHOF, RPL+, Rl-RPL, and ML-RPL by margins of 13%, 10%, 8%, and 5%, respectively.

### 5.3. Barcelona Scenario

**Figure 8:**
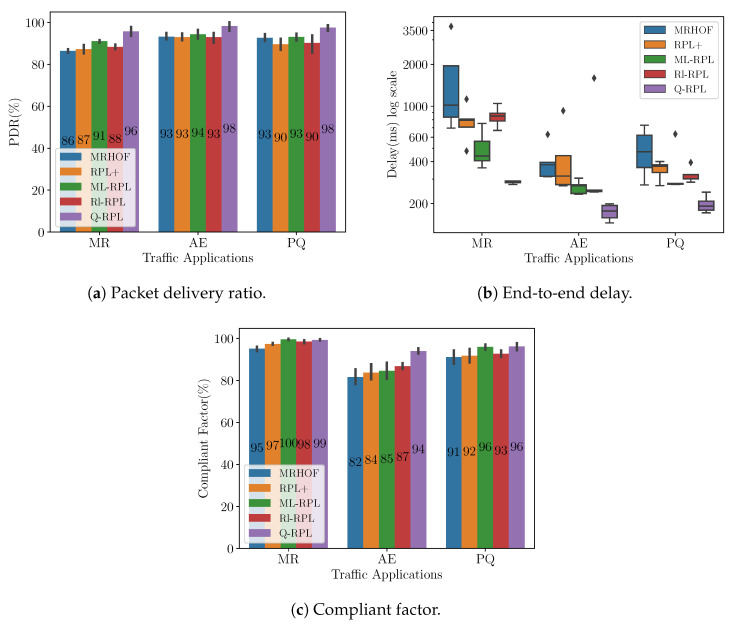
Performance metrics in the Barcelona scenario under traffic load 1.

We extended our testing to an AMI deployment in Barcelona, Spain, to further validate the efficacy of our routing protocol in diverse real-world scenarios. Distinct from the Montreal scenario, the Barcelona deployment, as noted in Section 5.1, consists of 355 smart meters. Moreover, the distribution of the smart meters is more centered around the collector. Figure 8a displays the PDR in the Barcelona scenario for traffic load 1. Q-RPL is observed to outperform the other routing variants across the three applications considered. Most notably, Q-RPL achieves a 10% higher PDR than MRHOF in the MR application, and an 8% greater PDR compared to RPL+ and Rl-RPL in the PQ application. Additionally, Q-RPL surpasses ML-RPL, its nearest competitor in terms of performance, by margins of 5%, 4%, and 5% for MR, AE, and PQ traffic, respectively.

The packet delay for this experiment is shown in Figure 8b. The figure clearly illustrates the superior performance of Q-RPL over the other routing protocols across all traffic applications in terms of median delay and consistency. Particularly notable is its performance in the MR traffic scenario, where it maintains a median delay of 286 ms, significantly lower than MRHOF, Rl-RPL, RPL+, and ML-RPL. This trend is consistent across AE and PQ traffic, where Q-RPL again demonstrates the lowest median delays (177 ms and 192 ms, respectively) and minimal variability. Rl-RPL, RPL+, and ML-RPL show improvements in median delay and consistency over MRHOF but still do not match the efficiency and reliability exhibited by Q-RPL.

In terms of CF, which is depicted in Figure 8c, we can see the same pattern as in the Montreal scenario. For MR traffic, all the routing protocols are close in performance, but for AE traffic the difference in favor of Q-RPL is more notable. In this case, Q-RPL’s CF stands at 94%, outperforming Rl-RPL, ML-RPL, RPL+, and MRHOF by margins of 7%, 9%, 10%, and 12%, respectively. For PQ traffic, both Q-RPL and ML-RPL lead the performance with a CF of 96%, indicating their superior ability over MRHOF, RPL+, and Rl-RPL in meeting stringent time constraints in packet delivery.

**Figure 9:**
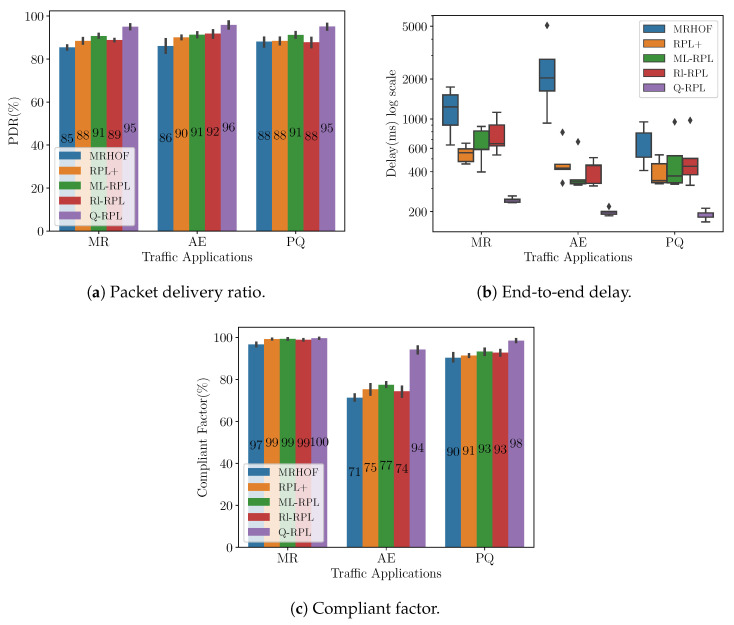
Performance metrics in the Barcelona scenario under traffic load 2.

Following our approach in the Montreal scenario, we further assessed the robustness of our routing protocols in the Barcelona deployment under increased traffic conditions. As illustrated in Figure 9a, the PDR in the Barcelona scenario with traffic load 2 again underscores the efficacy of Q-RPL. Q-RPL consistently outperforms the other routing variants across all application categories. For MR traffic, Q-RPL’s advantage over MRHOF peaks at a significant 10%, as for traffic load 1, while it holds a steady lead of 4% to 5% over ML-RPL throughout all the applications categories. Compared to RPL+, Q-RPL achieves a consistent performance edge of 6–7%, and compared to Rl-RPL, it shows an advantage of 6%, 4%, and 7% in the MR, AE, and PQ applications, respectively.

The end-to-end delay in the Barcelona scenario with traffic load 2 is shown in Figure 9b. It can be noticed from the figure that Q-RPL maintains a lower median delay, evident in its performance across the MR, AE, and PQ applications with median values of 246 ms, 199 ms, and 196 ms, respectively. In contrast, MRHOF shows a substantially higher median delay, particularly in the AE application, where it peaks at 2039 ms. RPL+, ML-RPL, and Rl-RPL record intermediate median delay values, positioned between Q-RPL and MRHOF across all traffic applications. These findings not only highlight the efficiency of Q-RPL but also reinforce its reliability under more challenging traffic conditions.

The enhanced performance in terms of end-to-end delay of our routing proposal is reflected in the CF metric, as depicted in Figure 9c. Q-RPL exhibits a CF of 100%, 94%, and 98% for MR, AE, and PQ traffic, respectively. A remarkable difference with respect to the other protocols is observed for AE traffic, where Q-RPL achieves up to a 17% improvement compared to ML-RPL, which is the next best performer.

### 5.4. General Discussion

In general, the simulation results from both the Montreal and Barcelona scenarios highlight Q-RPL’s superior performance in terms of PDR, network latency, and compliant factor compared to established protocols like MRHOF, RPL+, ML-RPL, and Rl-RPL.

The core strength of Q-RPL lies in its dynamic adaptability, achieved by integrating Q-learning into its decision-making process. This approach markedly differs from MRHOF’s reliance solely on the ETX metric and RPL+’s and ML-RPL’s dependence on preset rules and a trained ML model, respectively. When compared to Rl-RPL, another Q-learning-based protocol, Q-RPL demonstrates a more advanced and effective learning approach. Specifically, Rl-RPL’s approach of setting the learning rate in the Q-learning formula to one and the discount factor to zero led to overfitting to recent experiences and favored immediate rewards over long-term strategic routing. This approach made Rl-RPL behave more like a multimetric routing protocol rather than leveraging the full potential of Reinforcement Learning, which may explain its performance similarities to RPL+.

In contrast, Q-RPL distinguishes itself by continually learning from the network performance and adapting its parent selection in real-time with each packet transmission. This flexibility is crucial for managing the varied traffic patterns typical in AMIs.

**Figure 10:**
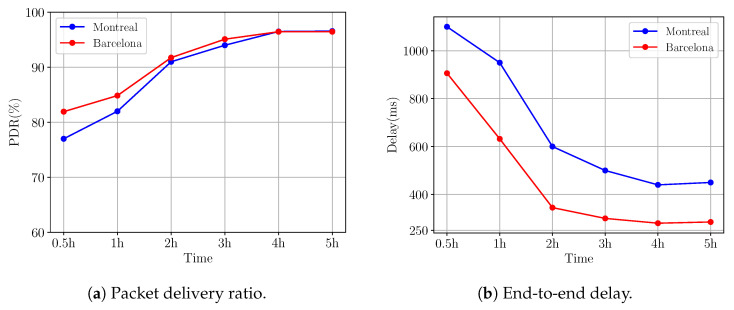
Average performance metrics progression in Montreal and Barcelona scenarios.

Figure 10a displays the evolution of the average PDR in the Montreal and Barcelona scenarios for Q-RPL under traffic load 1. This figure provides a clear illustration of the adaptive learning capability of the Q-RPL algorithm. During the initial stages, which are characterized by an exploratory approach, the PDR starts at a modest level. As the algorithm accumulates experience and refines its decision-making, improvements in PDR are observed. After simulating 2 h of network operation, a significant uptick in PDR performance is noted, with values plateauing at an optimal level. This sustained high performance highlights the efficiency of the Q-RPL’s learning mechanisms as they converge towards more effective routing choices over time.

Examining the average end-to-end delay as time progresses reveals an inverse correlation with the previously discussed PDR enhancement. Initially, the delay is notably higher in both scenarios, Figure 10b. In the Montreal scenario, the average delay in the first 30 min is 1100 ms, and exhibits a steep decline, stabilizing at around the 3 h mark of simulation time. Similarly, Barcelona shows an initial average delay in the first 30 min of 906 ms, which then decreases, leveling off at 285 ms after 3 h. This downward trend in delay underscores Q-RPL’s ability to reduce latency as the system progressively adapts to the network’s conditions.

**Figure 11:**
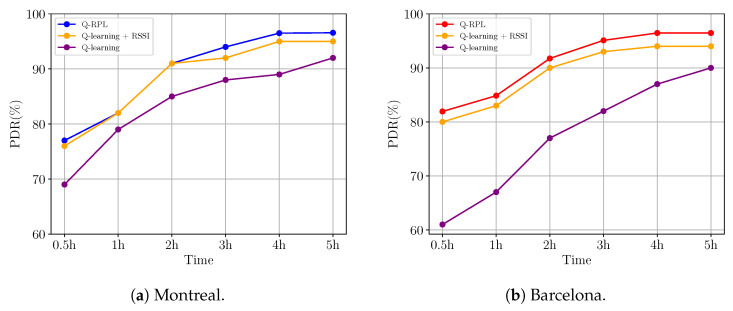
PDR progression in Montreal and Barcelona scenarios for different Q-RPL variants.

An important consideration in our approach was how to use some routing metrics to assist the Q-learning algorithm, specifically the RSSI and the ETX. We have illustrated the influence of these metrics on the PDR over time in Figure 11a,b. These figures clearly demonstrate how the inclusion of the RSSI metric speeds up the learning process. This speed-up was expected due to the metric’s role in constraining exploration to nodes with superior link quality. The difference observed between the protocol solely reliant on the Q-learning algorithm and the variant incorporating the RSSI metric is more pronounced in the Barcelona scenario, where the denser network of smart meters presents a wider array of routing choices. Thus, the narrowing of exploration space by the RSSI metric has more impact.

Figure 11a,b also depict the beneficial impact of integrating the ETX metric, used as a tiebreaker as described in Algorithm 2. The inclusion of ETX improves the algorithm’s performance in both scenarios. After 3 h of network operation, the data show a consistent performance improvement of 2–3% in the final version of Q-RPL compared to the version employing only Q-learning and RSSI. This enhancement underscores the value of ETX in refining decision-making, leading to more effective and reliable parent selection.

## 6. Technical/Critical Analysis and Recommendations for Deployment

In this section, we provide a comprehensive analysis of our research study, highlighting both its technical aspects and critical insights gained through the development process. We also offer recommendations for the deployment and usage of the Q-RPL protocol drawing from the challenges encountered during our study.

### 6.1. Technical Analysis

Our study aimed to improve the performance of the RPL routing protocol by integrating the Q-learning algorithm into it. Throughout the development phase, we encountered several technical challenges, the most notable being the construction and size management of the Q-table. Initially, we considered including metrics directly in the Q-table, which posed significant scalability issues. We opted for a simplified approach to overcome this challenge, maintaining the Q-table size proportional to the number of neighbors of the sending/forwarding node and integrating metric values into reward computations. This decision facilitated more manageable Q-tables while preserving essential information for decision-making within the protocol.

Additionally, we focused on fine-tuning the learning parameters. Special attention was given to the learning rate and the discount factor to balance the speed of convergence against the stability of the learning process. The learning rate was calibrated to control how quickly new information affected the Q-values, while the discount factor was adjusted to weigh the importance of future rewards. These parameters were optimized based on empirical results gathered from extensive simulations. This approach ensured that the learning process was neither too fast nor too slow, which could hinder timely convergence and adaptability in routing decisions. The selected settings demonstrated robust performance across various network conditions, significantly enhancing routing efficiency, as detailed in the results section.

### 6.2. Critical Analysis

A critical assessment of our approach reveals both strengths and limitations. For example, as previously mentioned, the simplification of the Q-table construction mitigated scalability concerns while maintaining the essential functionality of the Q-learning algorithm within the RPL protocol. However, this approach comes at the cost of rebuilding the Q-table whenever nodes are deployed in new scenarios or locations, thus requiring a repeat of the learning process. Consequently, this method sacrifices the potential benefits of transfer learning, where pre-trained Q-tables could serve as a starting point for further training in varied environments.

Another important consideration in Q-RPL is the balance between exploration and exploitation. Maintaining a degree of exploration is essential for discovering potentially better routing paths. However, excessive exploration might lead to instability and inefficiency, particularly in critical applications where consistent and reliable performance is paramount. For such applications or critical devices within the AMI network, it may be necessary to differentiate the exploration and exploitation policies. Integrating a QoS-aware strategy into Q-RPL could prevent critical data losses due to exploratory decisions, aligning network performance with operational priorities.

### 6.3. Recommendation for Deployment

Transitioning from simulation to real-world deployment of the Q-RPL protocol requires careful consideration to ensure it adapts effectively to actual network environments. The following is a structured approach for deploying this protocol:

- **Initial testbed trials:** Begin with small-scale experiments on actual hardware to understand how the protocol performs outside of simulation. This step is crucial for identifying any unforeseen issues that were not apparent during the simulation study.
- **Adaptation to hardware constraints:** This step may be necessary to ensure that the algorithm can operate efficiently without overwhelming device capabilities, maintaining optimal performance even within resource constraints. This step is important if initial evaluations indicate that the current learning algorithm exceeds the device’s operational limits.
- **Incremental deployment:** Gradually increases the scale of deployment while continuously monitoring system performance. This step allows for adjusting strategies in response to real-world challenges and complexities as they arise.
- **Performance monitoring and optimization:** Continuously collect and analyze performance data to optimize the protocol settings and adjustments.

## 7. Conclusions

The integration of Q-learning into the RPL protocol, named Q-RPL, represents a significant advancement in the adaptability and intelligence of routing decisions for Advanced Metering Infrastructure networks. By retaining the core functionalities of RPL and augmenting them with a learning-based approach for parent selection, we ensure both the reliability of traditional methods and the advantages of adaptive learning. The modifications in DIO messages for Q-value dissemination and the incorporation of ETX and RSSI metrics as auxiliary decision-making tools further refine the routing process.

The simulation results from both the Montreal and Barcelona scenarios consistently show the superior performance of Q-RPL compared to MRHOF, RPL+, ML-RPL, and Rl-RPL in diverse and dynamic AMI environments. In terms of packet delivery ratio, Q-RPL outperforms several benchmark routing protocols across various traffic applications. In the Montreal scenario under traffic load 1, Q-RPL demonstrates, for instance, a 12% improvement in the MR application compared to MRHOF, while for alarm events, it surpasses RPL+ by 6%, Rl-RPL by 7%, and MRHOF by 10%. A similar trend is observed using the Barcelona scenario, where Q-RPL achieves a 10% higher PDR than MRHOF in the MR application and an 8% greater PDR compared to RPL+ and Rl-RPL in the PQ application.

The end-to-end delay analysis reveals Q-RPL’s efficiency in maintaining low and consistent delay values across traffic applications, especially under an increased network load. In the Montreal scenario under traffic load 1, Q-RPL achieves a lower delay compared to the other routing variants of the order of 50–200 ms on average for MR traffic, and the difference in favor of Q-RPL becomes larger for AE and PQ traffic. This trend continues with traffic load 2, where Q-RPL maintains stable and low median delay values while other protocols such as MRHOF, RPL+, ML-RPL, and Rl-RPL experience increased variability. The Barcelona scenario exhibits similar results, with Q-RPL consistently showing lower median delay values and higher consistency compared to the MRHOF, RPL+, ML-RPL, and Rl-RPL protocols across all traffic applications for both traffic loads, demonstrating its adaptability to varying traffic conditions.

The compliant factor metric further highlights Q-RPL’s excellent performance in the area of service quality. Q-RPL, in the scenario of Montreal under traffic load 1, consistently achieves high CF values across all applications, outperforming other benchmark protocols such as MRHOF, RPL+, ML-RPL, and Rl-RPL, demonstrating its ability to meet specific application transit time requirements. Even under increased traffic load, Q-RPL maintains the highest CF for the AE and PQ applications, showing its robustness and adaptability. The results observed in the Barcelona scenario are consistent with those in Montreal, indicating that the Q-RPL’s CF remains consistently above 94% across all traffic applications and under traffic loads 1 and 2.

The adaptive learning capability of Q-RPL is shown in the evolution of average PDR and end-to-end delay over time. The algorithm starts with modest performance during the exploratory phase but steadily improves, reaching optimal PDR levels and minimizing delays. This dynamic adaptability is a key strength of Q-RPL, contributing to its high performance in changing network conditions.

In summary, the results obtained in this research strongly validate the promise of integrating Reinforcement Learning into communication routing protocols, leading to enhanced performance in AMI networks. Future work will focus on refining Q-RPL with Quality of Service (QoS) considerations and exploring other Reinforcement Learning models for further comparison. Specifically, we plan to investigate the potential of Deep Q-Networks (DQN) to manage high dimensional state spaces effectively and improve decision-making processes in dynamically changing network environments.

## Figures and Tables

**Table 1:** Comparative Analysis of RPL Enhancements.

Work	Key Contribution	Methodology	Performance Metrics	Limitations	Main Results
[24]	Uses multiple RPL instances to manage diverse traffic types in 6G/IoE health systems.	Standard RPL with hop count and ETX, tested in Cooja.	Improvements in packet delivery and highlighting significant latency reductions.	Standard RPL limitations may not meet all QoS needs in dynamic environments.	Enhanced diverse traffic management and efficiency in health applications.
[25]	Multi-topology RPL enhances QoS by novel parent selection in diverse traffic.	Multi-attribute decision-making for IoT parent selection.	Assesses delay, packet loss, PDR, throughput, queue loss ratio, routing overhead, and energy.	Limited simulation time may affect long-term adaptability in diverse network conditions	Improves QoS, shows scalability in large networks.
[26]	QWL-RPL targets heterogeneous traffic with dynamic queue and workload-based routing.	Utilizes queue length and MAC transmission rates for parent selection to minimize congestion.	Evaluates overhead, PRR, delay, and jitter under various loads.	Omission of link quality in routing risks suboptimal paths.	Reduces congestion significantly and improves PRR, delay, and jitter, but may overlook link quality.
[27]	WRF-RPL integrates energy and parent counts for balanced routing, enhancing network life and PDR.	Applies a weighted random forward method using energy and parent count for next-hop selection.	Evaluates network life, PDR, control overhead, and energy under high traffic.	Lacks link quality metrics, risking suboptimal paths; parent count may not reflect actual load.	Increases network life and PDR, risks uneven load distribution and inefficiencies.
[28]	RMP-RPL uses node mobility, connectivity, and ETX for reliable multi-parent selection.	Dynamic multi-path selection based on mobility, connectivity, and ETX.	Evaluates PDR, end-to-end delay, and overhead, showing notable improvements.	May increase congestion due to multiple simultaneous transmissions and is untested across variable traffic loads.	Enhances PDR and reduces delays, suitable for mision-critical applications with scalability concerns.
[31]	FL-HELR-OF uses fuzzy logic in a cross-layer architecture to dynamically select parents using hop count, energy, latency, and RSSI.	Fuzzy logic integrates multiple metrics into a cohesive routing decision framework.	Assesses PDR, latency, energy, overhead, and hop count, showing major improvements.	Requires complex setup and tuning, with potential scalability challenges.	Exceeds standard RPL in performance, improving reliability and efficiency in diverse IoT setups.
[34]	RPL+ uses Random Forest to refine parent selection by analyzing and weighting routing metrics.	Employs Random Forest for analyzing ETX, MAC losses, channel utilization, and throughput to optimize routing.	Evaluates PDR and end-to-end delay, noting improvements in network responsiveness.	Static weights in decision-making may limit adaptability to network changes.	Shows significant PDR improvements, enhancing reliability and efficiency compared to standard RPL.
[18]	ML-RPL uses CatBoost GBDT to enhance routing decisions via comprehensive metrics.	Model trained on smart meter data deployments, and predicts optimal routes based on probability.	Improvements in PDR and end-to-end delay demonstrate enhanced network efficiency.	Depends on training data quality, affecting adaptability in new scenarios.	Outperforms standard RPL and RPL+, especially in dynamic conditions.
[19]	GNB-RPL employs Gaussian Naive Bayes to optimize routing in smart grids for enhanced scalability.	Applies Gaussian Naive Bayes based on smart meter data from Montreal for routing decisions.	Shows marked improvements in packet delivery and reduced delays across varied loads.	Performance variability due to Naive Bayes’ simplistic assumptions about feature independence.	Enhances scalability and reduces training data needs, suitable for dynamic, large networks.
[20]	Analyzes supervised ML methods in RPL, focusing on scenario-dependent performance.	Assesses Catboost and Naive Bayes in varied scenarios for routing effectiveness.	Uses AUC to evaluate ML model predictions for routing success.	Effectiveness decreases in scenarios different to the training environment; requires frequent retraining.	Recommends exploring RL for adaptable, scenario-independent routing in dynamic settings.
[39]	Employs Q-learning in an RL-based protocol to optimize parent node selection in WSNs.	Uses Q-learning to dynamically choose the best parent based on network data and performance metrics.	Shows improvements in PDR, delay, and energy consumption over linear methods.	Needs more detail on reward function and concerns about scalability with periodic messages.	Surpasses traditional linear weighted methods, enhancing responsiveness and efficiency in dynamic WSNs.
[40]	Rl-RPL employs Q-learning to optimize IoT routing by addressing dynamic conditions and instant negative impacts of path selection.	Utilizes Q-learning to adapt to network changes, improving parent selection with real-time data.	Measures success delivery ratio, latency, energy, throughput, and data loss, noting major improvements.	Uniform weight in the reward function could impact decision accuracy; stability may reduce responsiveness after convergence.	Enhances service delivery, reduces delays, and boosts energy efficiency over previous methods.
[32]	Tabu-RPL employs Tabu Search to dynamically optimize routing in IoT, reducing network overhead.	Uses metaheuristic search based on ETX and residual energy to adapt routing to network conditions.	Evaluates network overhead, energy, PDR, and delay, showing significant reductions and improvements.	Details on overhead reduction and adaptability in diverse networks are not fully explained.	Achieves a 30% reduction in overhead, significantly enhancing energy efficiency and PDR.
[33]	EE-trickle modifies the trickle timer to boost energy efficiency and PDR in RPL.	Optimizes listening and transmission intervals to minimize energy use while maintaining performance.	Achieves notable reductions in energy per node and enhancements in PDR through simulations and testbeds.	May overlook challenges in dynamic environments, especially in link quality management.	Demonstrated enhanced energy efficiency and better PDR compared to the standard trickle method.
[41]	QFS-RPL combines Q-learning and FSR to optimize routing for mobile nodes in RPL.	Applies Q-learning and FSR for dynamic adjustments to network changes and mobile node management.	Boosts PDR, latency, throughput, and control overhead, while improving energy efficiency in mobile settings.	Performs similarly to standard RPL in static conditions and lacks extensive testing in dynamic environments.	Improves mobile performance by effectively managing mobility and load, but does not exceed standard RPL in static conditions.
[42]	ACTOR employs a UCB-based RL strategy for dynamic power management in RPL, boosting throughput in dense networks.	Uses UCB to dynamically adjust transmission power, optimizing power levels for better performance.	Significantly improves end-to-end delay, packet delivery, and energy consumption in dense networks.	Depends mainly on ETX for routing decisions, which may affect adaptability.	Enhances throughput and energy efficiency, stabilizes network topology with fewer parent switches.

**Table 2:** Q-table representation for node *i*.

State–Action Pair (s, a)	Q-Value
$\left(s_{1}^{i},a_{1}^{i}\right)$	$Q(s_{1}^{i},a_{1}^{i})$
⋮	⋮
$\left(s_{1}^{i},a_{k}^{i}\right)$	$Q(s_{1}^{i},a_{k}^{i})$
$\left(s_{n}^{i},a_{1}^{i}\right)$	$Q(s_{n}^{i},a_{1}^{i})$
⋮	⋮
$\left(s_{n}^{i},a_{k}^{i}\right)$	$Q(s_{n}^{i},a_{k}^{i})$

**Table 3:** Simulation Settings.

Network simulator	OMNeT++ v6.0.1 & INET Framework v4.4.1
Simulation runs	10/per scenario/per traffic load
Simulation time	5 h
Smart meters	200 (Montreal), 355 (Barcelona)
Collectors	1 (Montreal), 1 (Barcelona)
Channel characteristics	Path loss, $\alpha_{p}$ = 3.6
	Shadowing, Lognormal, σ = 7.4
PHY Layer	Standard, 802.15.4g
	Frequency band, 2.4 GHz
	Transmission rate, 115 Kbps
	Transmission power, 14 dBm
	Reception sensitivity, −100 dBm
	Energy detection, −90 dBm
	Min interference power, −120 dBm
MAC Layer	Standard, 802.15.4g
	Operation mode, Mesh
	ACK, Enable
	Max re-transmission, 3
	Backoff procedure, Exponential
	Min backoff exponent, 3
	Max backoff exponent, 8
Learning Layer	Learning rate, α = 0.3
	Discount factor, γ = 0.6
	ϵ = 1, $\epsilon_{min}$ = 0.05, $\epsilon_{decay}$ = 0.95
Packet size & sending interval	Application dependent, according to Table 4.

**Table 4:** SG applications transmitted over each scenario.

Traffic Load	Applications	Sending Period	Payload (Bytes)	Percentage of Meters
	Meter reading (MR)	Every 1 h	400	100%
1	Alarm events (AE)	Every 1 h	278	25%
	Power Quality (PQ)	Every 1 h	278	25%
	Meter reading (MR)	Every 30 min	400	100%
2	Alarm events (AEs)	Every 1 h	278	50%
	Power Quality (PQ)	Every 1 h	278	50%

**Table 5:** Targeted network transit time for each application.

Application	Network Transit Time
MR	2000 ms
AEs	500 ms
PQ	750 ms

## Data Availability

Data are contained within the article.

## Abbreviations

The following abbreviations are used in this manuscript:

AEs	Alarm Events
AMI	Advanced Metering Infrastructure
AUC	Area Under the Curve
CF	Compliant Factor
CTP	Collection Tree Protocol
DAO	Destination Advertisement Object
DAP	Data Aggregation Points
DIO	DAG information Object
DIS	DODAG Information Solicitation
DODAG	Destination-Oriented Directed Acyclic Graph
ETX	Expected Transmission Count
EWMA	Exponential Weighted Moving Average
GBDT	Gradient Boosted Decision Tree
GPSR	Geographic Routing Protocol
HWMP	Hybrid Wireless Mesh Protocol
HYDRO	Hybrid Routing Protocol
IoT	Internet of Things
LLN	Low-Power and Lossy Network
LOADng	Lightweight On-Demand Ad hoc Distance-vector Routing
	Protocol–Next-Generation
MAC	MAC Access Control
ML	Machine Learning
MR	Meter Reading
MRHOF	Minimum Rank with Hysteresis Objective Function
OLSR	Optimized Link State Routing Protocol
PDR	Packet Delivery Ratio
PQ	Power Quality
PRR	Packet Reception Ratio
QoS	Quality of Service
RF	Random Forest
RL	Reinforcement Learning
RPL	Routing Protocol for Low-Power and Lossy Networks
RSSI	Received Signal Strength Indicator
SGs	Smart Grids
UCB	Upper Confidence Bound
Wi-SUNs	Wireless Smart Utility Networks
WRF-RPL	Weighted Random Forward RPL
WSNs	Wireless Sensor Networks

## References

[1] (2021). IEEE Draft Standard for Information Technology-Telecommunications and Information Exchange between Systems-Local and Metropolitan Area Networks-Specific Requirements-Part 11: Wireless LAN Medium Access Control (MAC) and Physical Layer (PHY) Specifications-Amendment 10: Mesh Networking.

[2] (2011). IEEE Standard for Local and Metropolitan Area Networks-Part 15.4: Low-Rate Wireless Personal Area Networks (LR-WPANs) Amendment 3: Physical Layer (PHY) Specifications for Low-Data-Rate, Wireless, Smart Metering Utility Networks.

[3] Harada H., Mizutani K., Fujiwara J., Mochizuki K., Obata K., Okumura R. (2017). IEEE 802.15. 4g based Wi-SUN communication systems. IEICE Trans. Commun..

[4] Chang K.H., Mason B. (2012). The IEEE 802.15. 4g standard for smart metering utility networks. Proceedings of the 2012 IEEE Third International Conference on Smart Grid Communications (SmartGridComm).

[5] Karp B., Kung H.T. GPSR: Greedy Perimeter Stateless Routing for Wireless Networks. Proceedings of the 6th Annual International Conference on Mobile Computing and Networking.

[6] Fonseca R., Gnawali O., Jamieson K., Kim S., Levis P., Woo A. (2006). The collection tree protocol (CTP). TinyOS TEP.

[7] Clausen T., Dearlove C., Jacquet P., Herberg U. (2014). The Optimized Link State Routing Protocol Version 2.

[8] Winter T., Thubert P., Brandt A., Hui J., Kelsey R., Levis P., Pister K., Struik R., Vasseur J.P. (2012). IPv6 Routing Protocol for Low-Power and Lossy Networks.

[9] Dawson-Haggerty S., Tavakoli A., Culler D. (2010). Hydro: A hybrid routing protocol for low-power and lossy networks. Proceedings of the 2010 First IEEE International Conference on Smart Grid Communications.

[10] Clausen T., Yi J., Herberg U. (2017). Lightweight on-demand ad hoc distance-vector routing-next generation (LOADng): Protocol, extension, and applicability. Comput. Netw..

[11] Joshi A., Bahr M. (2006). HWMP specification. IEEE P802.

[12] Darabkh K.A., Al-Akhras M., Zomot J.N., Atiquzzaman M. (2022). RPL routing protocol over IoT: A comprehensive survey, recent advances, insights, bibliometric analysis, recommendations, and future directions. J. Netw. Comput. Appl..

[13] Iyer G., Agrawal P., Monnerie E., Cardozo R.S. (2011). Performance analysis of wireless mesh routing protocols for smart utility networks. Proceedings of the 2011 IEEE International Conference on Smart Grid Communications (SmartGridComm).

[14] Iyer G., Agrawal P., Cardozo R.S. (2013). Performance comparison of routing protocols over smart utility networks: A simulation study. Proceedings of the 2013 IEEE Globecom Workshops (GC Wkshps).

[15] Ho Q.D., Gao Y., Rajalingham G., Le-Ngoc T. (2014). Performance and applicability of candidate routing protocols for smart grid’s wireless mesh neighbor area networks. Proceedings of the 2014 IEEE International Conference on Communications (ICC).

[16] Ghaleb B., Al-Dubai A.Y., Ekonomou E., Alsarhan A., Nasser Y., Mackenzie L.M., Boukerche A. (2019). A Survey of Limitations and Enhancements of the IPv6 Routing Protocol for Low-Power and Lossy Networks: A Focus on Core Operations. IEEE Commun. Surv. Tutorials.

[17] Lamaazi H., Benamar N. (2020). A comprehensive survey on enhancements and limitations of the RPL protocol: A focus on the objective function. Hoc Netw..

[18] Santos C.L.D., Mezher A.M., León J.P.A., Barrera J.C., Guerra E.C., Meng J. (2023). ML-RPL: Machine Learning-based routing protocol for Wireless Smart Grid Networks. IEEE Access.

[19] Mezher A.M., Dueñas Santos C.L., Rebollo-Monedero D., Cárdenas-Barrera J., Aguilar Igartua M., Meng J., Castillo Guerra E. GNB-RPL: Gaussian Naïve Bayes for RPL Routing Protocol in Smart Grid Communications. Proceedings of the 19th ACM International Symposium on QoS and Security for Wireless and Mobile Networks.

[20] Mezher A.M., Dueñas Santos C.L., Astudillo Leon J.P., Cárdenas-Barrera J., Meng J., Castillo Guerra E. Are ML Models Scenario-Independent in Enhancing Routing Efficiency for Smart Grid Networks?. Proceedings of the Int’l ACM Symposium on Performance Evaluation of Wireless Ad Hoc, Sensor, & Ubiquitous Networks.

[21] Pister K., Dejean N., Barthel D. (2012). Routing Metrics Used for Path Calculation in Low-Power and Lossy Networks.

[22] Thubert E.P. (2012). Objective Function Zero for the Routing Protocol for Low-Power and Lossy Networks (RPL).

[23] Gnawali P.L.O. (2012). The Minimum Rank with Hysteresis Objective Function.

[24] Mardini W., Aljawarneh S., Al-Abdi A. (2021). Using Multiple RPL Instances to Enhance the Performance of New 6G and Internet of Everything (6G/IoE)-Based Healthcare Monitoring Systems. Mob. Netw. Appl..

[25] Bhandari K.S., Ra I.H., Cho G. (2020). Multi-Topology Based QoS-Differentiation in RPL for Internet of Things Applications. IEEE Access.

[26] Musaddiq A., Zikria Y.B., Zulqarnain, Kim S.W. (2020). Routing protocol for Low-Power and Lossy Networks for heterogeneous traffic network. Eurasip J. Wirel. Commun. Netw..

[27] Acevedo P.D., Jabba D., Sanmartin P., Valle S., Nino-Ruiz E.D. (2021). WRF-RPL: Weighted Random Forward RPL for High Traffic and Energy Demanding Scenarios. IEEE Access.

[28] Mishra S.N., Khatua M. (2022). Achieving Hard Reliability in RPL for Mission-Critical IoT Applications. Proceedings of the 2022 IEEE 8th World Forum on Internet of Things (WF-IoT).

[29] Gaddour O., Koubǎa A., Baccour N., Abid M. OF-FL: QoS-aware fuzzy logic objective function for the RPL routing protocol. Proceedings of the 2014 12th International Symposium on Modeling and Optimization in Mobile, Ad Hoc, and Wireless Networks, WiOpt 2014.

[30] Harshavardhana T.G., Vineeth B.S., Anand S.V., Hegde M. Power control and cross-layer design of RPL objective function for low power and lossy networks. Proceedings of the 2018 10th International Conference on Communication Systems and Networks, COMSNETS 2018.

[31] Darabkh K.A., Al-Akhras M., Ala’F K., Jafar I.F., Jubair F. (2022). An innovative RPL objective function for broad range of IoT domains utilizing fuzzy logic and multiple metrics. Expert Syst. Appl..

[32] Prajapati V.K., Sharma T., Awasthi L.K. (2024). Data Dissemination Framework for Optimizing Overhead in IoT-Enabled Systems Using Tabu-RPL. SN Comput. Sci..

[33] Shetty S.P., Shetty M., Kishore V., Shetty P. (2024). Trickle timer modification for RPL in Internet of things. Soft Comput..

[34] Duenas Santos C.L., Astudillo León J.P., Mezher A.M., Cardenas Barrera J., Meng J., Castillo Guerra E. RPL+: An Improved Parent Selection Strategy for RPL in Wireless Smart Grid Networks. Proceedings of the 19th ACM International Symposium on Performance Evaluation of Wireless Ad Hoc, Sensor, & Ubiquitous Networks.

[35] Raschka S., Mirjalili V. (2017). Python Machine Learning: Machine Learning and Deep Learning with Python, Scikit-Learn, and TensorFlow.

[36] Sun Y., Peng M., Zhou Y., Huang Y., Mao S. (2019). Application of machine learning in wireless networks: Key techniques and open issues. IEEE Commun. Surv. Tutorials.

[37] Ridwan M.A., Radzi N.A.M., Abdullah F., Jalil Y. (2021). Applications of machine learning in networking: A survey of current issues and future challenges. IEEE Access.

[38] Tang F., Mao B., Kawamoto Y., Kato N. (2021). Survey on machine learning for intelligent end-to-end communication toward 6G: From network access, routing to traffic control and streaming adaption. IEEE Commun. Surv. Tutorials.

[39] Kim B.S., Suh B., Seo I.J., Lee H.B., Gong J.S., Kim K.I. (2023). An Enhanced Tree Routing Based on Reinforcement Learning in Wireless Sensor Networks. Sensors.

[40] Zahedy N., Barekatain B., Quintana A.A. (2023). RI-RPL: A new high-quality RPL-based routing protocol using Q-learning algorithm. J. Supercomput..

[41] Alilou M., Babazadeh Sangar A., Majidzadeh K., Masdari M. (2024). QFS-RPL: Mobility and energy aware multi path routing protocol for the internet of mobile things data transfer infrastructures. Telecommun. Syst..

[42] Rabet I., Fotouhi H., Alves M., Vahabi M., Björkman M. (2024). ACTOR: Adaptive Control of Transmission Power in RPL. Sensors.

[43] Sutton R.S., Barto A.G. (1998). Reinforcement Learning: An Introduction.

[44] Raschka S., Liu Y.H., Mirjalili V., Dzhulgakov D. (2022). Machine Learning with PyTorch and Scikit-Learn: Develop Machine Learning and Deep Learning Models with Python.

[45] (2020). IEEE Standard for Low-Rate Wireless Networks.

[46] Kim H.S., Cho H., Kim H., Bahk S. (2017). DT-RPL: Diverse bidirectional traffic delivery through RPL routing protocol in low power and lossy networks. Comput. Netw..

[47] OMNeT++ Discrete Event Simulator. https://omnetpp.org/.

[48] Adday G.H., Subramaniam S.K., Zukarnain Z.A., Samian N. (2024). Investigating and Analyzing Simulation Tools of Wireless Sensor Networks: A Comprehensive Survey. IEEE Access.

[49] The ns-3 Network Simulator Project. ns-3 Network Simulator. https://www.nsnam.org/.

[50] Bartolozzi L., Pecorella T., Fantacci R. ns-3 RPL module: IPv6 routing protocol for low power and lossy networks. Proceedings of the 5th International ICST Conference on Simulation Tools and Techniques.

[51] Chen Y.b., Hou K.M., Chanet J.P., El Gholami K. A RPL based Adaptive and Scalable Data-collection Protocol module for NS-3 simulation platform. Proceedings of the NICST 2103 New Information Communication Science and Technology for Sustainable Development: France-China International Workshop.

[52] El Ghomali K., Elkamoun N., Hou K.M., Chen Y., Chanet J.P., Li J.J. A new WPAN Model for NS-3 simulator. Proceedings of the NICST’2103 New Information Communication Science and Technology for Sustainable Development: France-China International Workshop.

[53] Nagai Y., Guo J., Orlik P., Sumi T., Rolfe B.A., Mineno H. (2021). Sub-1 ghz frequency band wireless coexistence for the internet of things. IEEE Access.

[54] Leon J.P.A., Rico-Novella F.J., De La Cruz Llopis L.J. (2020). Predictive Traffic Control and Differentiation on Smart Grid Neighborhood Area Networks. IEEE Access.

[55] León J.P.A., Santos C.L.D., Mezher A.M., Barrera J.C., Meng J., Guerra E.C. (2023). Exploring the potential, limitations, and future directions of wireless technologies in smart grid networks: A comparative analysis. Comput. Netw..

